# Meniscus position and size in knees with versus without structural knee osteoarthritis progression: data from the osteoarthritis initiative

**DOI:** 10.1007/s00256-021-03911-8

**Published:** 2021-09-30

**Authors:** Kalpana Sharma, Felix Eckstein, Wolfgang Wirth, Katja Emmanuel

**Affiliations:** 1 Department of Imaging & Functional Musculoskeletal Research, Institute of Anatomy & Cell Biology, Paracelsus Medical University Salzburg & Nuremberg, Salzburg, Austria; 2 Department of Anatomy, Kathmandu University School of Medical Sciences, Dhulikhel, Nepal; 3 Ludwig Boltzmann Institute for Arthritis and Rehabilitation, Paracelsus Medical University, Salzburg, Austria; 4 Chondrometrics, GmbH, Freilassing, Germany; 5 Department of Orthopedics and Traumatology, Paracelsus Medical University, Salzburg, Austria

**Keywords:** Meniscus, Extrusion, Knee osteoarthritis, Magnetic resonance imaging, Progression

## Abstract

**Objective:**

To explore whether and which quantitative 3D measures of medial and/or lateral meniscus position and size are associated with subsequent medial femorotibial structural progression of knee osteoarthritis and to determine the correlation between central slice and total meniscus measures.

**Materials and Methods:**

Knees with radiographic osteoarthritis from Osteoarthritis Initiative participants with longitudinal medial MRI-based cartilage thickness and radiographic joint space width (JSW) loss over 12 months were selected. These 37 structural progressor knees (64.7 ± 8.0y, 30.2 ± 4.6 kg/m^2^, 35% men) were matched 1:1 to 37 non-progressor knees (64.6 ± 9.8y, 30.2 ± 4.4 kg/m^2^, 35% men) without cartilage thickness or JSW loss. Quantitative measures of meniscus position and size were computed from manual segmentations of coronal baseline MRIs. Cohen’s D was used as measure of effect size.

**Results:**

Maximum extrusion distance of the total medial meniscus and mean extrusion in the central 5 and in the central slice were greater for progressor than non-progressor knees (Cohen’s D 0.58–0.66). No significant differences were observed for medial tibial coverage or mean extrusion (entire meniscus). Among medial meniscus morphology measures, only mean height differed between progressor vs non-progressor knees (Cohen’s D 0.40). Among lateral meniscus measures, height and volume were greater in progressor vs. non-progressor knees (Cohen’s D 0.46–0.83). Mean extrusion measures were highly correlated between the entire meniscus and the central (*r* = 0.88) or the central 5 (*r* = 0.93) slices.

**Conclusions:**

3D maximum and central medial meniscus extrusion may serve as predictors for subsequent structural progression. Central meniscus extrusion measures could substitute 3D extrusion measurement across the entire meniscus.

## Introduction

Menisci distribute the load in the femorotibial joint [1], have a lubricative function, and are effective in reducing the stress on the articular cartilage during joint mobility [2]. Meniscal damage and extrusion are common in older individuals, even in those without knee symptoms or radiographic OA [3]. The cause for the extrusion has been attributed to the disruption of circumferential collagen bundle fibers in the meniscus [4] and particularly to a posterior root tear [5].

The menisci are considered of central importance for knee joint health [6]. Meniscal damage has been suggested to be a local risk factor for structural progression [7–10] and to be associated with subsequent knee replacement [11]. Surgical removal of the meniscus after injury was reported to be a significant risk factor for developing radiographic OA [12]. Quantitative (3D) measurement technology of meniscus position and size from MRI [13] has been shown to be reliable [14] and to be associated with development of incident radiographic OA [15], knee pain [16], and subsequent knee replacement [11].

Although knees with radiographic joint space narrowing (JSN) have been reported to show less tibial coverage and greater meniscus extrusion than contralateral knees without JSN [17], no study has yet explored which of the quantitative measures of medial meniscus position and morphology are most strongly related to subsequent structural progression, by comparing these measures in knees with radiographic knee OA with vs. without subsequent structural progression. Further, it has not been studied whether, and if yes to what extent, lateral meniscus measures are related to medial femorotibial progression.

The primary purpose of the current study was to explore whether quantitative measures of medial and/or lateral meniscus position and morphology differ between knees with and without subsequent medial femorotibial progression, and which of the medial or lateral meniscus 3D measures are most strongly associated with structural progression.

A secondary purpose of the study was to explore to what extent quantitative meniscus measures obtained in a central coronal slice, or in the central 5 coronal slices, correlate with those obtained in the entire meniscus, and whether these measures differentiate equally well between progressors and non-progressors. Such central slice measures may be less prone to partial volume effects in the anterior and posterior parts of the meniscus and also require less segmentation time than the analysis of the entire meniscus.

## Materials and methods

### Study design and sample selection

All clinical and imaging data were drawn from the Osteoarthritis Initiative (OAI) database http://www.oai.ucsf.edu/. General inclusion criteria for the OAI have been published and are publicly available http://oai.epi-ucsf.org/datarelease/ (https://nda.nih.gov/oai/, ClinicalTrials.gov Identifier: NCT00080171) [18]. Informed consent was obtained from all 4796 participants and the study was approved by the local ethics committees.

The study included participants that were previously selected for investigating the association between thigh anatomical muscle cross-sectional areas with structural knee OA progression [19]. In brief, OAI knees with and without medial structural progression were selected from a sample of 725 OAI participants with longitudinal data on change in cartilage thickness obtained from 3 Tesla MRI [20] and in radiographic joint space width (JSW) obtained from fixed-flexion radiography [21] as described previously [19, 22]. Cartilage thickness change was assessed from a double oblique coronal fast low angle shot (FLASH) MRI sequence that was acquired in the right knees of the OAI participants [19, 20], who were graded as Kellgren & Lawrence grade (KLG) 2–4 according to the site radiographic readings [23].

The smallest detectable change (SDC) method [24] was used to identify knees with definite progression. The SDC thresholds for MRI-based change in cartilage thickness were computed from baseline and follow-up test–retest data from the OAI pilot study [25] and were 102 µm for cartilage thickness loss in the medial femorotibial compartment and 92 µm for cartilage thickness loss in the lateral femorotibial compartment. To confirm the structural progression observed by MRI using another independent method, change in radiographic minimum JSW (minJSW) was used to ensure apparent changes in cartilage loss (SDC threshold from repeated measurements of same radiographs: 328 µm). Progressor knees were defined as those with a reduction in both medial femorotibial cartilage thickness and medial minJSW exceeding both of the above thresholds. These were matched to non-progressor knees, which were defined as those without a reduction in medial femorotibial cartilage thickness, medial minJSW, as well as lateral femorotibial cartilage thickness exceeding the above thresholds.

**Fig. 1:**
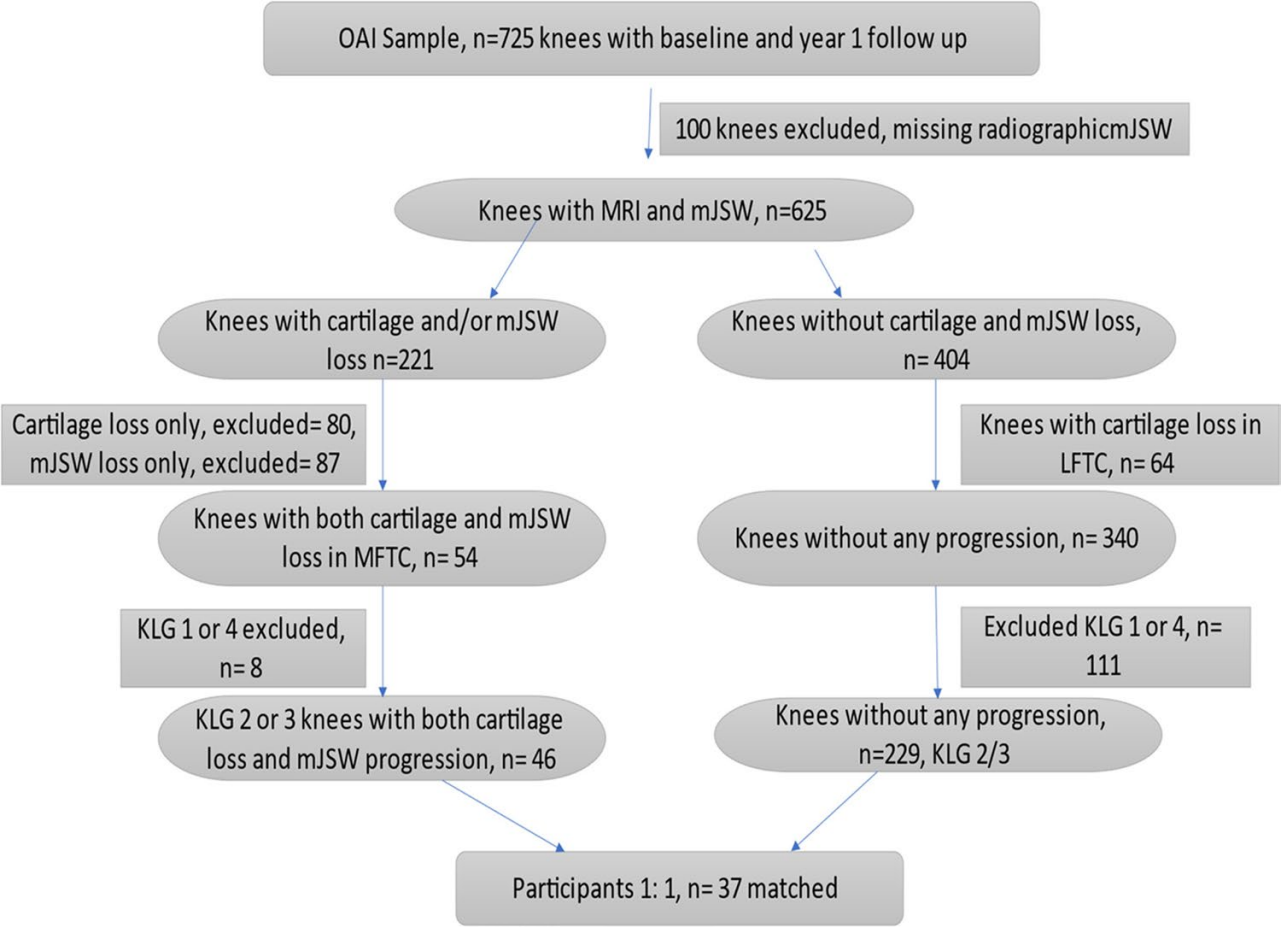
Flow chart showing the selection process of knees with structural progression of both cartilage loss and minimum joint space width (mJSW) in the medial femorotibial compartment (MFTC) and control knees without progression of mJSW and cartilage loss between the baseline and year 1 follow-up. The case and control knees were matched by sex, BMI, baseline Kellgren-Lawrence grade (KLG 2/3), and pain. No knees had to be excluded because of inadequate image quality or non-adherence to MRI protocol parameters

Of the 725 knees, 100 had to be excluded due to missing minJSW measurements. Of the remaining 625 knees, 54 knees qualified as progressor knees by exceeding the SDC thresholds for both cartilage loss and minJSW loss in the medial compartment and 340 knees qualified as non-progressor knees by not exceeding one of the SDC thresholds explained above (Fig. 1). After excluding knees without definite radiographic OA (KLG < 2) or with end-stage radiographic OA (KLG4) at baseline according to the OAI central readings, 46 of the 54 progressor and 229 of the 340 non-progressor knees were considered for matching of progressor and non-progressor knees.

Knees with and without structural progression were matched 1:1 by the same sex, baseline KLG (2 or 3), body height ± 3 cm, BMI ± 5 kg/m^2^, and Western Ontario and McMaster Universities Osteoarthritis Index (WOMAC) pain scores ± 5 (scale from 0 to 20). Of the 46 case and 229 control knees fulfilling the mentioned criteria, 37 progressors could be matched to 37 non-progressors.

### MR imaging

MRIs were acquired at the OAI clinical sites using 3 Tesla Magnetom Trio magnets (Siemens Erlangen, Germany) and quadrature transmit-receive knee coils (USA Instruments, Aurora, OH) [26]. The coronal multi-planar reconstructions of the sagittal double echo steady state sequence with water excitation (DESSwe: reconstructed slice thickness = 1.5 mm, in-plane resolution 0.37 mm × 0.7 mm, interpolated to 0.37 mm × 0.37 mm) were used for manual segmentation of the menisci, because this sequence has been shown to yield acceptable inter-observer reliability and good agreement with measurements made from a coronal intermediate-weighted turbo spin echo (IW-TSE) sequence [14]. The advantage of the DESS is, however, that it provides greater spatial resolution and better delineation of the tibial plateau cartilage surface area than the IW-TSE and also has been validated for accurately depicting the tibial cartilage [27]. The intra-observer reproducibility of 3D quantitative meniscus extrusion measurements and the inter-observer reproducibility of 3D quantitative meniscus assessments have been published before [14, 28].

### Meniscus segmentation and quantitative analysis

**Fig. 2:**
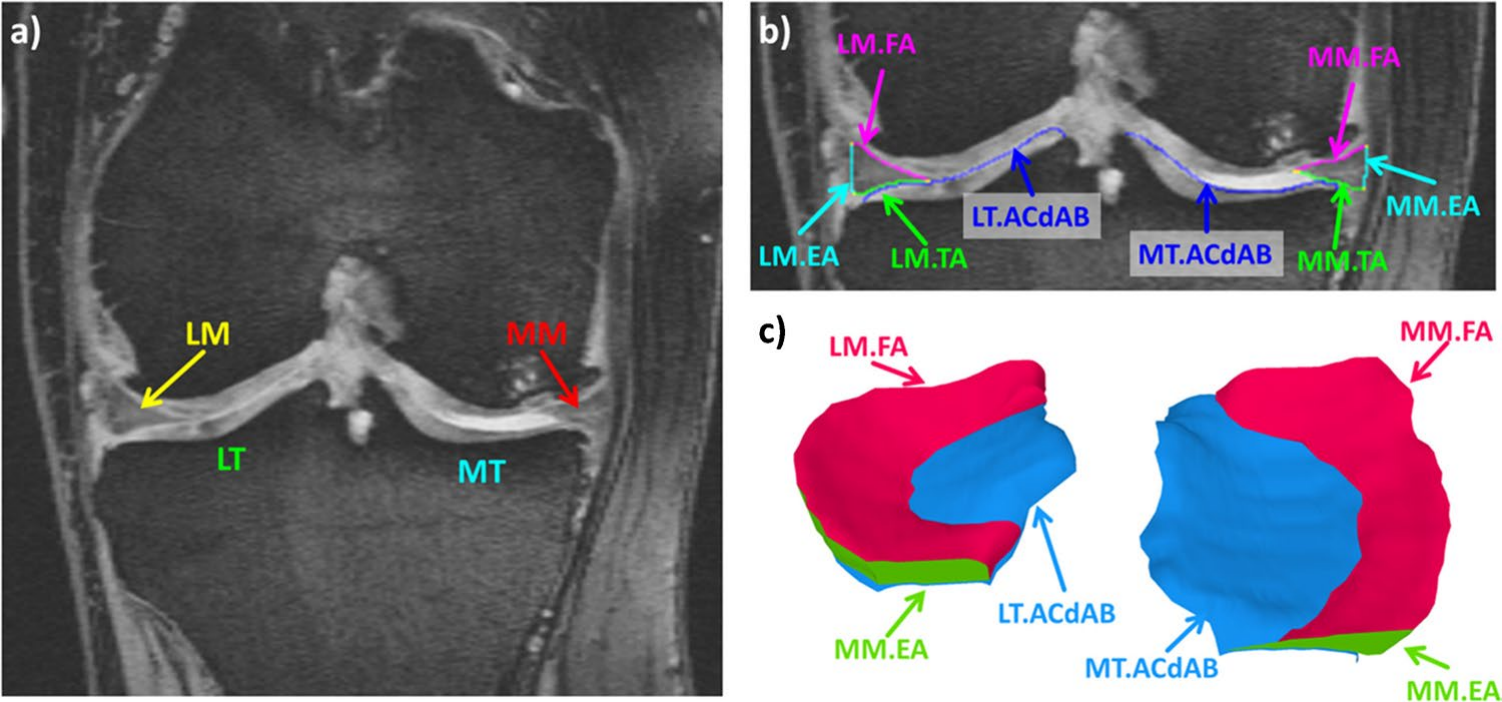
**a** Coronal reconstruction of the sagittal DESS showing the medial meniscus (MM) & lateral meniscus (LM) and the cartilage of the medial tibia (MT) & lateral tibia (LT). **b** Detailed view of the femorotibial joint showing the segmentation of the MM & LM areas (TA: Tibial area, FA: Femoral area & EA: External area) and the cartilage surface area (ACdAB) of the MT and LT. **c** 3D reconstruction showing the LM and MM from anterior/superior

All images underwent initial quality control to ensure adequate image quality and adherence to the MRI protocol before the manual segmentation of the medial and lateral tibial plateau area (i.e., the area of cartilage surface, including denuded areas of subchondral bone = ACdAB [13]), as well as the segmentation of the medial and lateral meniscus (tibial, femoral and external surface) by a single experienced operator (> 3 years and > 500 segmented knees) was performed. Segmentation was performed using dedicated image analysis software (Chondrometrics GmbH, Freilassing, Germany); it started anteriorly and ended posteriorly in the first/last image in which both the tibial cartilage and the menisci could be reliably identified (Fig. 2). Internally, the borders of the menisci were defined by the internal margin of the cartilage surfaces of the medial tibia, because these are continuous with the transverse and menisco-femoral ligaments and because no intrinsic anatomical demarcation could be used to separate these structures. Meniscus position and morphology measures were computed from the manual segmentations using a software developed specifically for the purpose of quantitative meniscus analyses [13]. Meniscus position measures included the position of the meniscus relative to the tibial plateau (percentage of tibial plateau covered by the meniscus), the mean 3D extrusion of the meniscus (distance between the external margin of the tibial plateau area and that of the tibial meniscus area) for the entire meniscus, the central, and the central 5 slices, the maximum extrusion distance, and the area of the tibial meniscus surface not covering the tibial plateau (in percent of the meniscus tibial surface). Meniscus morphology measures included the volume, the mean and maximal meniscus height (thickness), and the mean meniscus width for the entire meniscus, the central, and the central 5 slices [13].

### Statistical analysis

Mean values and standard deviations (SD) were determined for all quantitative measures of the medial and lateral meniscus morphology and position for progressors and non-progressors separately. Differences between progressors and non-progressors were evaluated as the mean difference and 95% confidence intervals from paired comparisons. *P*-values were computed using paired *t*-tests, Cohen’s D was used as effect size measure. Pearson’s correlation was used to determine the strength and direction of the relationship of parameters between the entire meniscus vs the central slice, and the central 5 slices.

## Results

### Sample description

The 37 matched progressors and non-progressors (13 male, 24 female) had a similar age (64.7 ± 8.0 years vs. 64.6 ± 9.8 years, *p* = 0.98), height (165.6 ± 7.9 cm vs 165.6 ± 7.7 cm, *p* = 0.94), BMI (30.2 ± 4.6 kg/m^2^ vs 30.2 ± 4.4 kg/m^2^, *p* = 0.94), and anatomical axis alignment measured from fixed-flexion X-rays (− 6.1 ± 2.9° vs − 5.7 ± 2.5°, *p* = 0.40), whereas the WOMAC pain scores tended to be greater in the progressor than non-progressor knees (3.5 ± 3.8 vs 2.8 ± 3.3, *p* = 0.04, Table 1). Arthroscopic surgery to repair or cut away torn meniscus or cartilage was reported by 7 progressors and 6 non-progressors for their right knees.

**Table 1:** Baseline characteristics of the matched progressors and non-progressors (each *n* = 37)

		Progressors (Cases)	Non-progressors (Controls)
		Mean ± SD	Mean ± SD
Age	(years)	64.7 ± 8.0	64.6 ± 9.8
BMI	(kg/m^2^)	30.2 ± 4.6	30.2 ± 4.4
Height	(cm)	165.6 ± 7.9	165.6 ± 7.7
WOMAC	(1…..20)	3.5 ± 3.8	2.8 ± 3.3
Anatomic axis	(°)	− 6.1 ± 2.9°	− 5.7 ± 2.5°
		*N*	*N*
Sex	Male	13	13
	Female	24	24
KLG	2	21	21
	3	16	16
Med JSN	0	7	14
	1	17	11
	2	13	12
Lat JSN	0	32	28
	1	2	4
	2	3	5

Anatomic axis or femorotibial angle measurements from fixed-flexion X-rays were available for 36 of the progressor and 35 of the non-progressor knees, more negative values indicate a tendency toward varus alignment

*WOMAC* Western Ontario and McMaster Universities Osteoarthritis Index, *KLG* Kellgren & Lawrence grade, *JSN* joint space narrowing according to the OARSI atlas, *SD* Standard deviation

Baseline cartilage thickness and minJSW in the medial femorotibial compartment were comparable between progressors and non-progressors (Table 2), whereas baseline cartilage thickness in the lateral compartment was greater in progressors than non-progressors (Table 2). Over the one year follow-up, progressor knees showed a significant loss in medial compartment cartilage thickness and minJSW that was also greater than the loss observed in non-progressors. Only little change was observed in lateral compartment cartilage thickness in both progressors and non-progressors (not statistically significant)*,* although the difference between groups reached statistical significance (Table 2).

**Table 2:** Minimum joint space width and cartilage thickness loss in the *n* = 37 progressors and the *n* = 37 non-progressors

	Progressors	Non-progressors	Difference	*P* value
	Mean ± SD	Mean ± SD	Mean (95% CIs)	
Baseline				
minJSW (mm)	3.9 ± 1.4	3.8 ± 1.3	0.1 (− 0.4, 0.6)	0.62
MFTC (mm)	3.2 ± 0.6	3.3 ± 0.6	− 0.1 (− 0.3, 0.1)	0.36
LFTC (mm)	3.8 ± 0.5	3.6 ± 0.5	0.3 (0.1, 0.5)	0.01
Change from baseline to 1-year follow-up				
minJSW (µm)	− 1052 ± 788	88 ± 259	− 1140 (− 1445, − 841)	< 0.01
MFTC (µm)	− 254 ± 165	21 ± 78	− 275 (− 346, − 209)	< 0.01
LFTC (µm)	− 39 ± 144	19 ± 59	− 57 (− 112, 0)	0.02

*minJSW* minimum radiographic joint space width in the medial compartment, *MFTC* Medial femorotibial compartment cartilage thickness, *LFTC* Lateral femorotibial compartment cartilage thickness, *SD* Standard deviation, 95% *CIs* 95% confidence intervals

### Quantitative measures of the medial meniscus position and morphology

The mean extrusion distance of the entire meniscus was not observed to differ between progressors and non-progressors (0.4 mm, 95% CI: (− 0.1 mm, 0.9 mm), Cohen’s D: 0.38, *p* = 0.09; Table 3). The maximum extrusion distance of the total meniscus was, however, greater for the progressors than for the non-progressors (0.8 mm, 95% CI: (0.2 mm, 1.4 mm), Cohen’s D: 0.66, *p* ≤ 0.01, Table 3). Also, the mean extrusion was greater in progressors and non-progressors when measured in the central 5 slices (0.8 mm, 95% CI: (0.2 mm, 1.5 mm), Cohen’s D: 0.58, *p* < 0.01) and the central slice (1.0 mm, 95% CI: (0.3 mm, 1.6 mm), Cohen’s D: 0.62, *p* < 0.01, Table 3). No differences were observed for the percentage of the tibial plateau covered by the medial meniscus (− 6.9%, 95% CI: (2.5%, − 0.17%), Cohen’s D: − 0.17, *p* = 0.35), meniscus width (− 0.2 mm, 95% CI: (− 0.8 mm, 0.3 mm), Cohen’s D: − 0.14, *p* = 0.39), and meniscus volume (0.1 ml, 95% CI: (− 0.1 ml, 0.3 ml), Cohen’s D: 0.12, *p* = 0.40). Mean medial meniscus height was greater for progressors than for non-progressors (0.2 mm, 95% CI: (0.0 mm, 0.3 mm), Cohen’s D: 0.40, *p* = 0.02, Table 3). The mean extrusion in the central 5 slices and the central slice showed a greater effect size (Cohen’s D 0.58 / 0.62) than mean extrusion in the entire meniscus (0.38).

**Table 3:** Medial meniscus position & size in *n* = 37 progressors vs *n* = 37 non-progressors

	Cases		Controls		Difference Cases vs Controls				
	Mean	SD	Mean	SD	Mean	95% CI		Cohen's D	*P*
Extrusion area (%)	30.4	13.4	26.7	17.3	3.7	− 2.1	9.5	0.24	0.20
Mean extrusion distance (mm)	2.6	1.1	2.2	1.2	0.4	− 0.1	0.9	0.38	0.09
Max. extrusion distance (mm)	4.8	1.3	4.0	1.1	0.8	0.2	1.4	0.66	< 0.01
Mean extrusion 5 central slices (mm)	3.3	1.4	2.5	1.5	0.8	0.2	1.5	0.58	< 0.01
Mean extrusion central slice (mm)	3.4	1.5	2.4	1.6	1.0	0.3	1.6	0.62	< 0.01
Tibial plateau coverage (%)	34.6	12.5	36.8	13.5	− 2.2	− 6.9	2.5	− 0.17	0.35
Width mean total (mm)	7.9	1.7	8.2	1.8	− 0.2	− 0.8	0.3	− 0.14	0.39
Height mean (mm)	2.8	0.4	2.6	0.4	0.2	0.0	0.3	0.40	0.02
Volume (ml)	1.9	0.7	1.8	0.7	0.1	− 0.1	0.3	0.12	0.40

*SD* Standard deviation, 95% *CIs* 95% confidence intervals

### Quantitative measures of lateral meniscus position and morphology

Measures of lateral meniscus position as well as lateral meniscus width did not differ between progressor and non-progressor knees (Table 4). Meniscus height was, however, greater in progressor than non-progressor knees (0.2 mm, 95% CI: (0.1 mm, 0.4 mm), *p* < 0.01) with an effect size that exceeded those observed for medial compartment measures (Cohen’s D; 0.83). Progressor knees also had a greater lateral meniscus volume than non-progressor knees (0.2 ml, 95% CI: (0.0 ml, 0.4 ml), Cohen’s D: 0.46, *p* = 0.03).

**Table 4:** Lateral meniscus position and size in *n* = 37 progressors vs *n* = 37 non-progressors

	Cases		Controls		Difference Cases vs Controls				
	Mean	SD	Mean	SD	Mean	95% CI		Cohen's D	*P*
Extrusion area (%)	7.8	7.3	8.6	10.6	− 0.7	− 5.3	3.9	− 0.08	0.75
Mean extrusion distance (mm)	− 0.3	1.2	− 0.4	1.2	0.1	− 0.6	0.7	0.06	0.83
Max. extrusion distance (mm)	2.0	1.5	1.8	1.3	0.1	− 0.5	0.8	0.09	0.68
Mean extrusion 5 central slices (mm)	0.1	1.4	− 0.2	1.2	0.3	− 0.4	1.0	0.22	0.41
Mean extrusion central slice (mm)	0.0	1.4	− 0.2	1.2	0.2	− 0.5	0.8	0.13	0.60
Tibial plateau coverage (%)	54.1	8.3	51.7	11.6	2.4	− 2.6	7.3	0.23	0.34
Width mean total (mm)	8.4	1.1	8.0	1.3	0.4	− 0.1	1.0	0.34	0.12
Height mean (mm)	2.7	0.3	2.4	0.3	0.2	0.1	0.4	0.83	< 0.01
Volume (ml)	1.8	0.4	1.6	0.5	0.2	0.0	0.4	0.46	0.03

*SD* Standard deviation, 95% *CIs* 95% confidence intervals

### Correlation between measurement of the central slice(s) and the entire meniscus

**Fig. 3:**
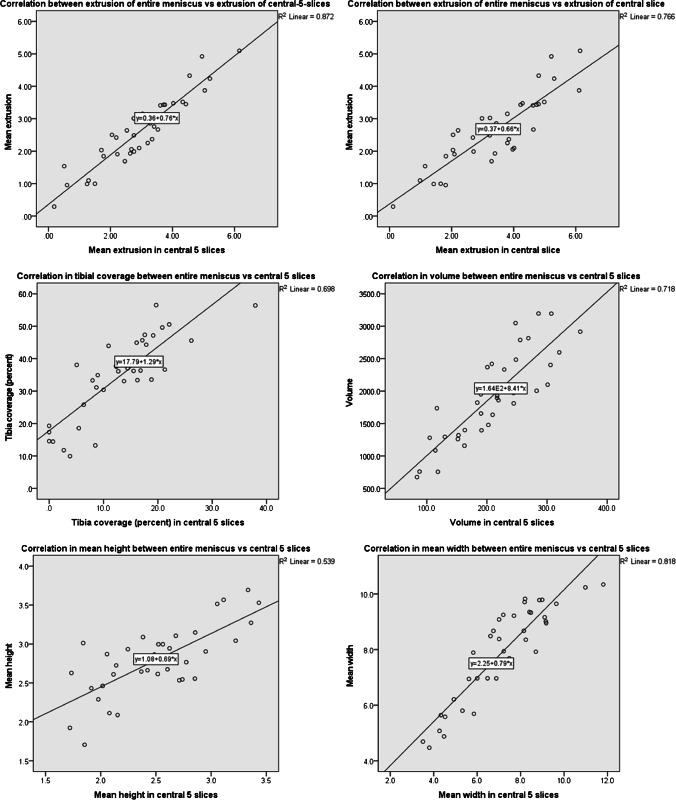
Graphs showing the correlation between medial meniscus measures obtained from the central slice or the central 5 slices vs. measures obtained across the entire medial meniscus

Across case and control knees, mean extrusion in the entire meniscus was highly correlated with mean extrusion in the central 5 slices (*r* = 0.93) as well as with mean extrusion in the central slice (*r* = 0.88, Table 5, Fig. 3). Positive correlations between the entire meniscus and the central 5 slices were also observed for the tibial plateau coverage (*r* = 0.84), the volume (*r* = 0.85), the height (*r* = 0.73), and the width (*r* = 0.90, Table 5; Fig. 3).

**Table 5:** Correlation and linear regression of measures obtained from the entire medial meniscus vs measures obtained from the central slice and the central 5 slices

	Pearson correlation (*r*)	Linear regression (r^2^%)
Mean extrusion distance: entire vs. central 5 slices	0.93	87
Mean extrusion distance: entire vs. central slice	0.88	76
Tibia plateau coverage (%): entire vs. central 5 slices	0.84	69
Volume: entire vs. central 5 slices	0.85	71
Mean height: entire vs. central 5 slices	0.73	53
Mean width: entire vs. central 5 slices	0.90	81

## Discussion

In our study, we investigated whether (and which) quantitative measures of medial and lateral meniscus position and morphology differ between knees with and without subsequent medial femorotibial structural progression using a matched study design and explored whether quantitative measures obtained from the center of the meniscus can serve as surrogate for quantitative measures obtained from the entire meniscus. The results from our study identified several (but not all) meniscus position measures to differ between knees with vs. without subsequent medial compartment structural progression, whereas only one of the medial meniscus morphology measures (height) differed between knees with vs. without subsequent progression. Among the lateral meniscus measures, both height and volume differed between progressor than non-progressor knees, with greater values observed in progressor than non-progressor knees. Quantitative measures obtained in the central 5 or the central slice of the medial meniscus were highly correlated with the respective measures obtained from the entire meniscus and the differences between progressor and non-progressor knees were more pronounced for mean extrusion in the central 5 or the central slice than for mean extrusion in the entire medial meniscus.

Meniscus extrusion and damage have been reported to be important risk factors for the development and progression of knee OA, but most of these studies relied on semi-quantitative scorings of meniscus pathology and meniscus extrusion [29–31]. Quantitative 3D measures of meniscus position and morphology have been developed to provide a comprehensive, quantitative picture that not only includes the meniscus position but also the meniscus morphology. The reproducibility of the methodology has been validated and quantitative 3D meniscus measures have been reported to be associated with relevant outcomes such as development of incident radiographic OA [15] and presence of pain [16]. More recently, change in meniscus measures has also been shown to be associated with subsequent knee replacement surgery [11].

The model of accelerated progression used in the current study has been previously used to study differences in thigh muscle cross-sectional areas [19], thigh adipose tissue [32], and cartilage T2 [22]. In contrast to the findings of the current study, none of these previous measures was, however, observed to be a strong predictor of subsequent structural progression. This is despite the tremendous loss in cartilage thickness observed in progressor knees, which even exceeded the loss in knees in the year before knee replacement surgery [33]. The meniscus, therefore, seems to play a more immediate role for subsequent progression than thigh muscle and adipose tissue and to be more predictive of subsequent structural progression than cartilage T2 measures.

The results from the current study extend findings from previous studies. Bloecker et al. reported a significantly greater extrusion and a significantly reduced tibia plateau coverage in knees with JSN than in contralateral knees without JSN [17]. Hunter et al. studied the contribution of meniscus extrusion to radiographic JSN using a quantitative 2D methodology and reported change in meniscal position to account for a substantial proportion of change in radiographic JSW [34]. Later, Roth et al. investigated change in 3D meniscus measures in knees before joint replacement surgery and reported meniscus measures to not only show a significant change but also to provide independent information in explaining the variance of change in radiographic JSW [35]. Although these studies already suggested meniscus measures to be associated with structural progression measured from radiographs, none of these studies investigated specifically whether quantitative meniscus measures may predict subsequent structural progression and some of these previous studies focused on the direct contribution of meniscus measures to change in radiographic JSW.

The results from the current study showed medial meniscus extrusion to be greater in knees with vs. without subsequent structural progression, indicating a potential role as predictor for subsequent structural progression in knees with radiographic OA. Across the entire meniscus, the difference did, however, only reach statistical significance for the maximum but not the mean extrusion. Knees with subsequent progression also showed a somewhat greater extrusion area and a somewhat reduced tibia plateau coverage when compared to non-progressor knees, but these differences did also not reach statistical significance. This is in line with findings from a previous study investigating the association between meniscus measures and incident radiographic OA [15], which suggested tibial coverage to be among the least informative measures and to be of less importance than meniscus extrusion. Whether the coverage of the tibia plateau by the meniscus is also comparable between progressor and non-progressor knees under dynamic, weight-bearing conditions is, however, not known, because analyses of the meniscus from MRI are limited to non-weight-bearing conditions.

Medial meniscus height was significantly greater in knees with than in knees without subsequent progression. This may be the consequence of the greater extrusion of the meniscus in progressor than non-progressor knees, because the greater extrusion results in greater parts of the meniscus positioned outside the joint space, where the meniscus shape adapts due to the constraints imposed by the joint capsule and ligaments. Interestingly, also the lateral meniscus height was significantly greater in knees with than in knees without subsequent progression and displayed the greatest effect size between progressor and non-progressor knees. This difference can, however, not be attributed to differences in extrusion, but might be explained by the greater lateral meniscus volume observed in progressor than non-progressor knees. Increased lateral meniscus height or volume should, however, not be considered as direct risk factors for medial compartment progression because the greater values observed in knees with subsequent medial compartment progression could also be due to preceding loss of lateral meniscus substance in knees without subsequent medial compartment progression.

The high correlation observed between meniscus measures obtained from the entire meniscus and from the central slices indicates that restricting the analysis to the center is a suitable replacement for measures obtained from the entire meniscus. Also, while differences in mean medial meniscus extrusion across the entire meniscus did not reach statistical difference between progressor and non-progressor knees, mean medial meniscus extrusion in the central 5 slices or the central slice was greater in progressor knees than non-progressor knees and showed a greater effect size than that observed for mean extrusion across the entire medial meniscus. An analysis of the central slices of the meniscus may, therefore, not only save segmentation time when compared to the analysis of the entire meniscus, but could also improve the sensitivity to differences between groups.

Differences in meniscus extrusion were observed although medial compartment cartilage thickness and radiographic JSW at baseline did not differ between the knees with vs without subsequent progression studied here. The results from the current study, therefore, suggest that quantitatively measured meniscus extrusion may be a valuable predictor for subsequent structural progression. Given the small sample size, the predictive value of meniscus extrusion still needs to be confirmed in larger studies. The association between meniscus extrusion and subsequent cartilage loss is, however, plausible given that the meniscus plays an important role in distributing loads and in balancing the incongruity of the femorotibial cartilages. Meniscus extrusion and damage have also been suggested to be local risk factors for cartilage loss. Chang et al. reported semi-quantitatively scored meniscus tears to be associated with cartilage thickness loss in adjacent cartilage subregions [7]. More recently, Bloecker et al. reported the effect of meniscus extrusion on cartilage thickness loss to be most pronounced for the external medial tibia subregion, the cartilage subregion most severely affected from medial–lateral extrusion [36]. Meniscus damage has also been reported to be associated with elevated superficial cartilage T2 relaxation times in adjacent tibial articular cartilage [37], indicating detrimental alterations in cartilage composition in the affected regions. In the same sample as studied here, we were, however, not able to observe differences in cartilage T2 relaxation times (or change therein) in the external medial cartilage subregion [22], the subregion most affected from central medial extrusion.

A limitation of the study is the small sample size. This can be attributed to the strict selection criteria that were applied to ensure to only select knees with definite loss in both MRI-based cartilage thickness and radiographic JSW as progressor knees. The sample size was, however, sufficiently large to observe statistically significant results between progressor and non-progressor knees and the combination of MRI and radiography thresholds ensured significant differences in structural progression between progressor and non-progressor knees. Another limitation of the study is that the coronal MRIs did not allow to assess the anterior and posterior extrusion of the menisci due to partial volume effects. Analyzing the anterior and posterior extrusion would have required to analyze sagittal MRIs of the same knees, which would have, in turn, precluded the assessment of extrusion in the body of the meniscus. Another potential limitation of this study is that the quantitative approach used here is not capable of assessing meniscus damage such as tears. These are, however, highly prevalent in knees with radiographic OA as well as in the general population [3]. Finally, one of the selection criteria (radiographic JSW) was not independent from the meniscus measures, as meniscus extrusion has been reported to contribute to JSN on radiographs [34, 38].

In conclusion, medial meniscus extrusion was significantly greater in knees with subsequent progression than in knees without subsequent progression while measures of tibia plateau coverage and most measures of medial meniscus morphology differed not significantly between progressor and non-progressor knees. Meniscus extrusion may, therefore, serve as potential predictor of subsequent structural progression in knees with radiographic OA, in particular when focusing on maximum extrusion or measures obtained from the central slice(s) of the meniscus. The high correlation between central meniscus measures and measures obtained from the entire meniscus indicate that central meniscus measures are suitable substitutes of entire meniscus measures that save analysis time and may be even more sensitive to between-group differences.
